# Supplementary material for: Designing of a next generation multiepitope based vaccine (MEV) against SARS-COV-2: Immunoinformatics and in silico approaches
Source: PLoS One. 2020 Dec 22;15(12):e0244176. doi: 10.1371/journal.pone.0244176 (PMC7755200; doi:10.1371/journal.pone.0244176)
Supplement: S2 Fig — Hydrogen bond interactions are highlighted with yellow color dotted lines. Numbering 1–11 is consistent with the Table 1 of main text. (DOCX) [file pone.0244176.s002.docx]

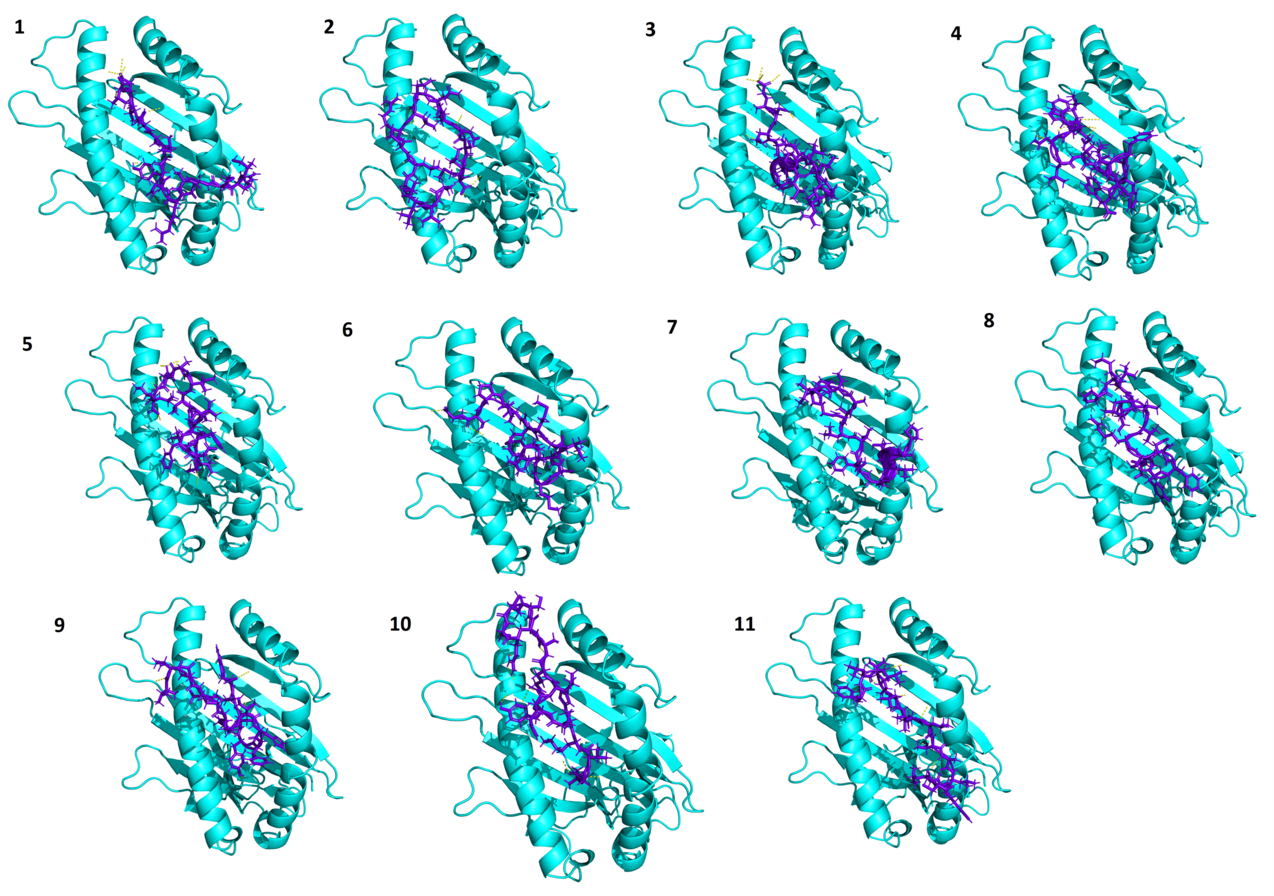


**Fig S2**. The 3D binding pattern of the selected 11 MHC Class II epitopes (purple blue) docked with HLA-B7 allele (cyan). Hydrogen bond interactions are highlighted with yellow color dotted lines. Numbering 1-11 is consistent with the Table 1 of main text.
