# Supplementary figures and images for: Designing of a next generation multiepitope based vaccine (MEV) against SARS-COV-2: Immunoinformatics and in silico approaches
Source: PLoS One. 2020 Dec 22;15(12):e0244176. doi: 10.1371/journal.pone.0244176 (PMC7755200; doi:10.1371/journal.pone.0244176)

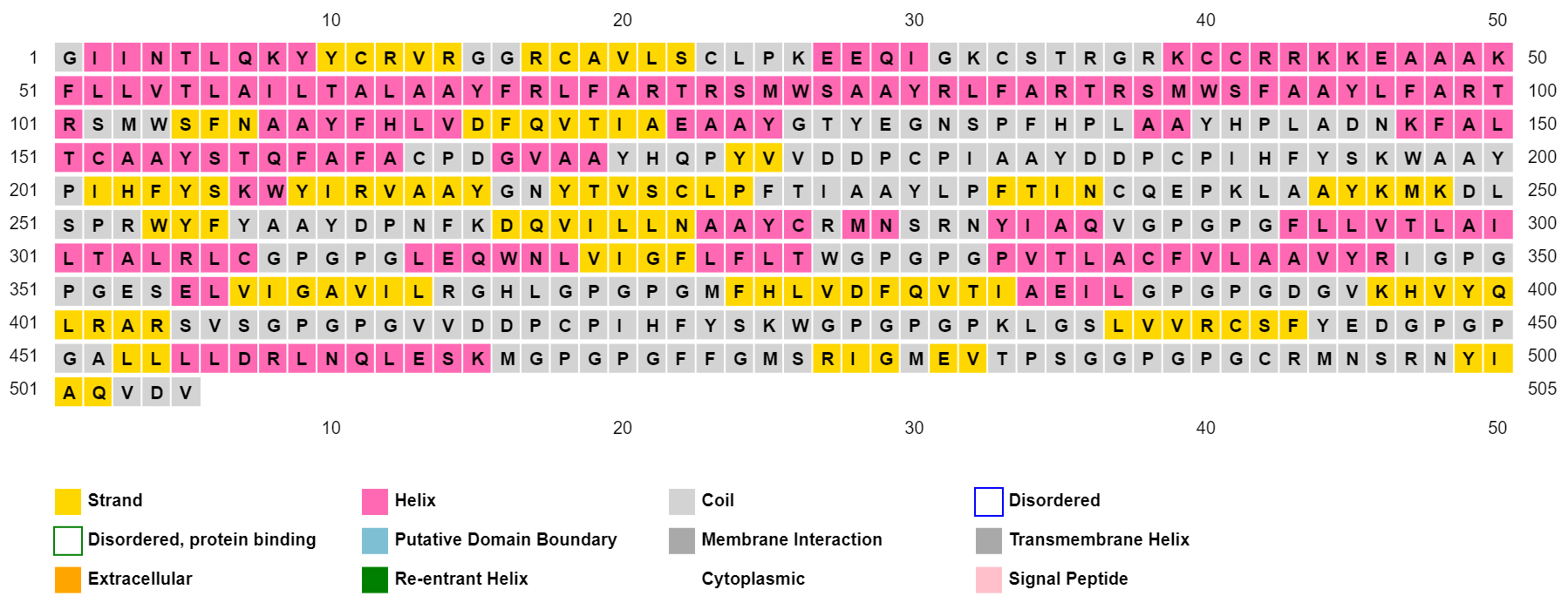


**Fig S3**. Secondary structure depiction of MEV construct.

Supplement: S3 Fig — (DOCX) [file pone.0244176.s003.docx]
