# Supplementary material for: Designing of a next generation multiepitope based vaccine (MEV) against SARS-COV-2: Immunoinformatics and in silico approaches
Source: PLoS One. 2020 Dec 22;15(12):e0244176. doi: 10.1371/journal.pone.0244176 (PMC7755200; doi:10.1371/journal.pone.0244176)
Supplement: S6 Table — Green color bold sequence at 5`site (N-terminal) is representing XhoI restriction enzyme site, while maroon color bold sequence at 3`site (C-terminal) is representing HindIII restriction enzyme site. (DOCX) [file pone.0244176.s009.docx]

Supplementary Table 6. Codon optimized nucleotide sequence of MEV construct for cloning in *E. coli* strain K12. Green color bold sequence at 5` site (N-terminal) is representing XhoI restriction enzyme site, while maroon color bold sequence at 3` site (C-terminal) is representing HindIII restriction enzyme site.

| **CTCGAG**GGTATCATCAACACCCTGCAGAAATACTACTGCCGTGTTCGTGGTGGTCG TTGCGCTGTTCTGTCTTGCCTGCCGAAAGAAGAACAGATCGGTAAATGCT CTACCCGTGGTCGTAAATGCTGCCGTCGTAAAAAAGAAGCTGCTGCTAAA TTCCTGCTGGTTACCCTGGCTATCCTGACCGCTCTGGCTGCTTACTTCCG TCTGTTCGCTCGTACCCGTTCTATGTGGTCTGCTGCTTACCGTCTGTTCG CTCGTACCCGTTCTATGTGGTCTTTCGCTGCTTACCTGTTCGCTCGTACC CGTTCTATGTGGTCTTTCAACGCTGCTTACTTCCACCTGGTTGACTTCCA GGTTACCATCGCTGAAGCTGCTTACGGTACCTACGAAGGTAACTCTCCGT TCCACCCGCTGGCTGCTTACCACCCGCTGGCTGACAACAAATTCGCTCTG ACCTGCGCTGCTTACTCTACCCAGTTCGCTTTCGCTTGCCCGGACGGTGT TGCTGCTTACCACCAGCCGTACGTTGTTGACGACCCGTGCCCGATCGCTG CTTACGACGACCCGTGCCCGATCCACTTCTACTCTAAATGGGCTGCTTAC CCGATCCACTTCTACTCTAAATGGTACATCCGTGTTGCTGCTTACGGCAA CTACACCGTGAGCTGCCTGCCGTTCACCATCGCTGCTTACCTGCCGTTCA CCATCAACTGCCAGGAACCGAAACTGGCTGCTTACAAAATGAAAGACCTG TCTCCGCGTTGGTACTTCTACGCTGCTTACGACCCGAACTTCAAAGACCA GGTTATCCTGCTGAACGCTGCTTACTGCCGTATGAACTCTCGTAACTACA TCGCTCAGGTTGGGCCGGGGCCGGGCTTCCTGCTGGTTACTCTGGCTATC CTGACCGCTCTGCGTCTGTGCGGTCCGGGTCCGGGTCTGGAACAGTGGAA CCTGGTTATCGGTTTCCTGTTCCTGACCTGGGGTCCGGGTCCGGGTCCGG TTACCCTGGCTTGCTTCGTTCTGGCTGCTGTTTACCGTATCGGTCCGGGT CCGGGTGAATCTGAACTGGTTATCGGTGCTGTTATCCTGCGTGGTCACCT GGGTCCGGGTCCGGGTATGTTCCACCTGGTTGACTTCCAGGTTACCATCG CTGAAATCCTGGGTCCGGGTCCGGGTGACGGCGTTAAACACGTGTACCAG CTGAGAGCGCGTTCTGTAAGCGGTCCGGGTCCGGGTGTTGTTGACGACCC GTGCCCGATCCACTTCTACTCTAAATGGGGTCCGGGTCCGGGTCCGAAAC TGGGTTCTCTGGTTGTTCGTTGCTCTTTCTACGAAGACGGTCCGGGTCCG GGTGCTCTGCTGCTGCTGGACCGTCTGAACCAGCTGGAATCTAAAATGGG GCCGGGGCCGGGCTTCTTCGGTATGTCGCGTATCGGTATGGAAGTTACCC CGTCTGGTGGTCCGGGTCCGGGTTGCCGTATGAACTCTCGTAACTACATC GCTCAGGTTGACGTT**AAGCTT** |
| --- |
